# Supplementary material for: K-RAS Acts as a Critical Regulator of CD44 to Promote the Invasiveness and Stemness of GBM in Response to Ionizing Radiation
Source: Int J Mol Sci. 2021 Oct 10;22(20):10923. doi: 10.3390/ijms222010923 (PMC8539357; doi:10.3390/ijms222010923)
Supplement: Supplementary file 1 [file ijms-22-10923-s001.zip › ijms-1320749-supplementary.pdf]

K-RAS Acts as a Critical Regulator of CD44 to Promote the Invasiveness and Stemness of GBM in Response to Ionizing Radiation

Yi Zhao [1,†], Jae-Hyeok Kang [1,†], Ki-Chun Yoo [1], Seok-Gu Kang [2], Hae-June Lee [3] and Su-Jae Lee [1,*]

1 Department of Life Science, Research Institute for Natural Sciences, Hanyang University, Seoul 04763, Korea; zhaoyi0924@gmail.com (Y.Z.); jaehyeok1121@gmail.com (J.-H.K.); vanity0706@gmail.com (K.-C.Y.)
2 Department of Neurosurgery, Brain Tumor Center, Severance Hospital, Yonsei University College of Medicine, Seoul 03722, Korea; seokgu9@yuhs.ac (S.-G.K)
3 Division of Radiation Effect, Korea Institute of Radiological and Medical Sciences, Seoul 01812, Korea; hjl22@kirams.re.kr
* Correspondence: sj0420@hanyang.ac.kr; Tel.: +82-2-2220-2557
† These authors contributed equally to this work.

Supplementary Information:

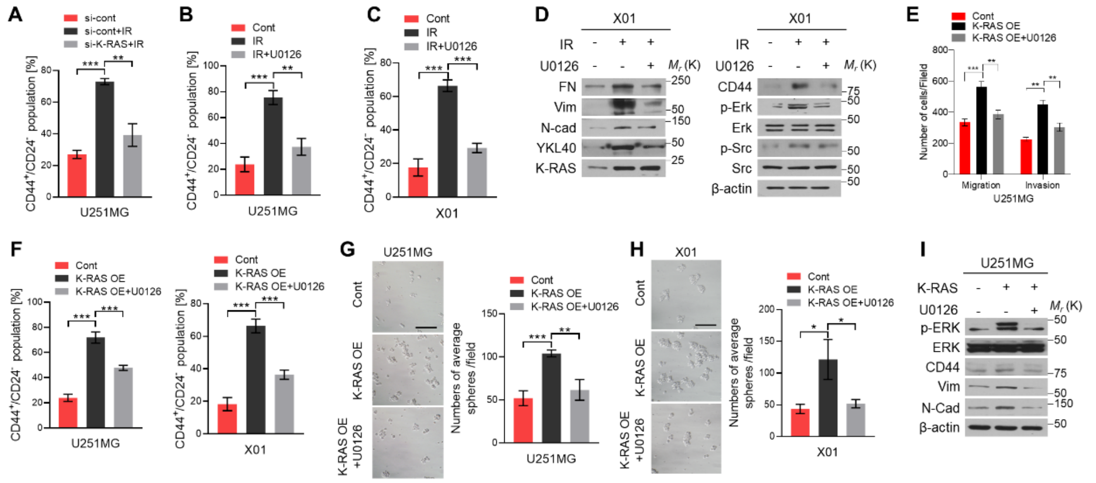

Figure S1. K-RAS/ERK/CD44 axis regulates IR-induced stemness in GBM cells. (**A**). FACS analysis of the percentage of CD44+/CD24- cells using the IR-treated U251MG cells with or without K-RAS expression. (**B–D**). FACS and western blotting analysis were performed to assess the cell stemness features and mesenchymal markers expression using the IR- treated U251MG and patient-derived X01 GBM cells, respectively, with or without the treatment of U0126. (**E**). Invasion assay were performed to assess the invasiveness in the K-RAS-overexpressing U251MG cells treated with or without U0126. (**F–H**). FACS analysis and sphere formation assay were used to measure the stemness features of K-RAS-overexpressing U251MG cells and patient-derived X01 GBM cells, respectively, with the treatment of U0126 or not. (**I**). Western blotting analysis of p-ERK, ERK, CD44, Vim, and N-Cad expression using the same condition samples of above. β-actin was used as a control for normalization of expression. ** $p < 0.001$, *** $p < 0.0001$, n.s., not significant. A two-tailed Student's t-test was used to compare data between two groups, and ANOVA was used for multiple comparisons.

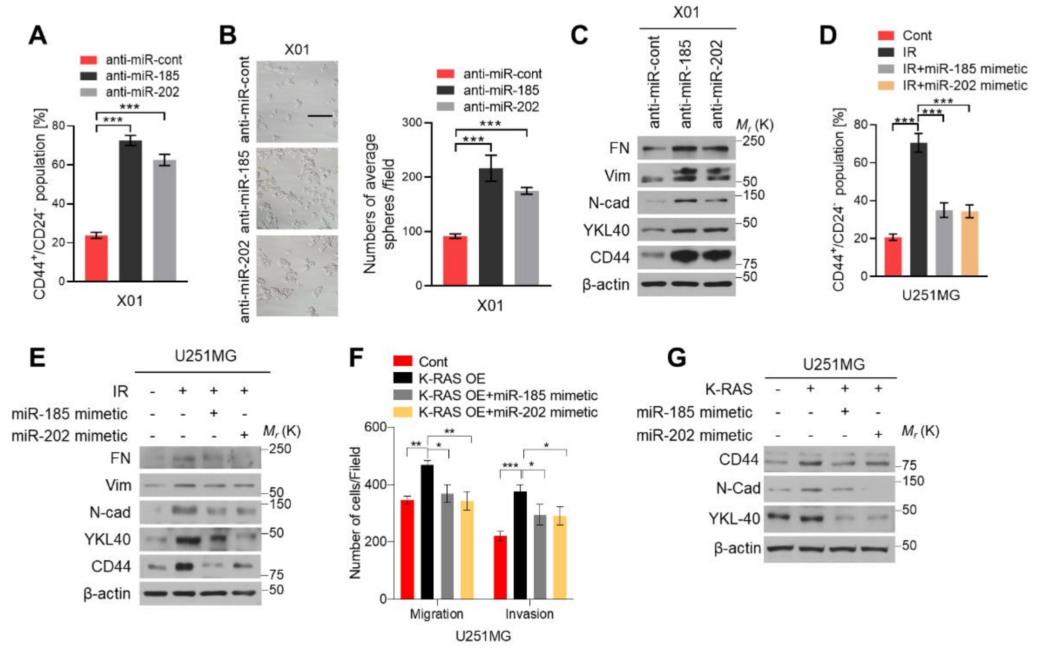

Figure S2. miR-185 and miR202 inhibit stemness of GBM by suppressing CD44 expression. (**A**,**B**)**.** FACS analysis of the percentage of CD44+/CD24- cells in patient-derived X01 GBM cells with the treatment of miR-185 or miR-202 inhibitor. (**C**)**.** Western blotting analysis of mesenchymal markers and CD44 expression using the same condition samples of above. (**D**,**E**). FACS and western blotting analysis were performed to assess the stemness features and the expression of mesenchymal markers in IR-treated U251MG cells, transfected with miR-185 or miR-202 mimetic. (**F**,**G**). Invasion and migration assay and western blotting analysis were performed to measure the invasiveness ability of cells and the mesenchymal markers expression in U251MG cells with overexpression of the K-RAS alone or together with miR-185 and/or miR-202 mimetic. β-actin was used as a control for normalization of expression. * $p < 0.05$, ** $p < 0.001$, *** $p < 0.0001$, n.s., not significant. A two-tailed Student's t-test was used to compare data between two groups, and ANOVA was used for multiple comparisons.

Table S1. The list of qRT-PCR primer sequences.

**Primer Name**	**Sequence (5′-3′)**	
Fibronectin	Sense:	ACCAACCTACGGATGACTCG
	Antisense:	GCTCATCATCTGGCCATTTT
CDH2	Sense:	GACAATGCCCCTCAAGTGTT
	Antisense:	CCATTAAGCCGAGTGATGGT
Vimentin	Sense:	GAACCAATGAGTCCCTGGAA
	Antisense:	TCCAGCAGCTTCCTGTAGGT
CD133	Sense	GCCACCGCTCTAGATACTGC
	Antisense	TGTTGTGATGGGCTTGTCAT
β-actin	Sense:	CATCCGCAAAGACCTGTACG
	Antisense:	CCTGCTTGCTGATCCACATC
